# Bacterial Chaperones CsgE and CsgC Differentially Modulate Human α-Synuclein Amyloid Formation *via* Transient Contacts

**DOI:** 10.1371/journal.pone.0140194

**Published:** 2015-10-14

**Authors:** Erik Chorell, Emma Andersson, Margery L. Evans, Neha Jain, Anna Götheson, Jörgen Åden, Matthew R. Chapman, Fredrik Almqvist, Pernilla Wittung-Stafshede

**Affiliations:** 1 Department of Chemistry, Umeå University, Umeå, Sweden; 2 Department of Molecular, Cellular and Developmental Biology, University of Michigan, Ann Arbor, Michigan, United States of America; 3 Umeå Centre for Microbial Research, Umeå University, Umeå, Sweden; University of Maryland School of Medicine, UNITED STATES

## Abstract

Amyloid formation is historically associated with cytotoxicity, but many organisms produce functional amyloid fibers (*e*.*g*., curli) as a normal part of cell biology. Two *E*. *coli* genes in the curli operon encode the chaperone-like proteins CsgC and CsgE that both can reduce *in vitro* amyloid formation by CsgA. CsgC was also found to arrest amyloid formation of the human amyloidogenic protein α-synuclein, which is involved in Parkinson’s disease. Here, we report that the inhibitory effects of CsgC arise due to transient interactions that promote the formation of spherical α-synuclein oligomers. We find that CsgE also modulates α-synuclein amyloid formation through transient contacts but, in contrast to CsgC, CsgE accelerates α-synuclein amyloid formation. Our results demonstrate the significance of transient protein interactions in amyloid regulation and emphasize that the same protein may inhibit one type of amyloid while accelerating another.

## Introduction

Amyloid fibrils are β-sheet rich protein structures associated with human neurodegenerative diseases, such as Alzheimer’s and Parkinson’s diseases [1]. The common structural element of these fibrils is the cross-β conformation; *i*.*e*., β-sheets that are packed perpendicular to the fiber axis. Most amyloid fibrils form via nucleation-dependent pathways that involve oligomeric, pre-fibrillar structures [2]. α-Synuclein is a 140-residue protein involved in Parkinson´s disease, a condition which affects 2% of the population older than 60 years [3]. Albeit synaptic signaling has been proposed, the exact function of α-synuclein is unknown. It is an intrinsically unstructured protein that adopts α-helical structure in the presence of vesicles or membranes [4]. Residues 60 to 95 in the α-synuclein primary sequence (the so called NAC region) constitute the primary aggregation-promoting region [4] whereas the acidic C-terminal part (96–140) tempers aggregation [5]. The α-synuclein fibrils accumulate in cytosolic inclusions called Lewy bodies found in brains of Parkinson’s disease patients [6].

Numerous studies have been pursued in order to find small-molecule and protein inhibitors of α-synuclein fiber formation as possible means to combat Parkinson’s disease. For many such cases, the *in vitro* end products are oligomers or aggregates that do not proceed to amyloid fibers. For example, selegiline causes nontoxic amorphous aggregates of α-synuclein [7], baicalein induces spherical α-synuclein oligomers [8], and rifampicin also stabilizes oligomers [9]. Little is known about the molecular mechanisms and driving forces underlying small molecule/protein interactions with α-synuclein. We recently demonstrated that designed peptidomimetic compounds based on a 2-pyridone scaffold modulated α-synuclein amyloid formation *in vitro* [10, 11]. Depending on precise chemical substitutions of the ring-fused 2-pyridone central fragment, molecules were discovered that either promoted or inhibited α-synuclein amyloid formation [10, 11].

Amyloid formation is not only associated with disease. Nature employs the amyloid structure for a number of functions [12, 13]. In particular, microbial functional amyloids are major components of the extracellular matrix that promotes biofilm formation and other community behaviors [12–14]. The amyloid-forming properties of *Escherichia coli* CsgA protein are among the most studied [14, 15]. The extracellular curli fibers, that are common components of bacterial biofilms, are composed of polymerized CsgA and are attached to the surface of the bacteria through membrane-bound CsgB which are believed to act as a “seed” for polymerization of CsgA [16, 17]. CsgA is an unstructured monomeric protein that rapidly forms amyloid fibrils both *in vivo* and *in vitro* [18, 19]. The expression of CsgA is tightly regulated and at least two gene products of the curli operon, CsgE and CsgC, was recently shown to act as periplasmic chaperones and prevent fibril formation in the cell prior to export [14, 20], as well as inhibit CsgA amyloid formation *in vitro* [21]. Whereas CsgC was effective at sub-stoichiometric amounts [20], CsgE required equal mixing with CsgA to block amyloid formation *in vitro* [21]. Moreover, exogenously added CsgE could inhibit curli-dependent biofilm formation in a pellicle biofilm assay [21]. CsgC is a monomeric protein of 110 residues with a β-sandwich fold [22]. The structure of the 129-residue CsgE protein has not been determined [14] although it was recently proposed to be a nonamer [23].

Here, we tested if CsgE could also modulate α-synuclein amyloid formation. Although we discovered that both bacterial chaperones affected α-synuclein via transient protein-protein interactions, the presence of CsgE, in sharp contrast to CsgC and to its effect on CsgA, resulted in promotion of α-synuclein amyloid formation *in vitro*.

## Materials and Methods

### Expression and purification of α-synuclein

The construct for human α-synuclein was ordered from GenScript (NJ, USA) and cloned into a pET-3a vector. The construct carries the gene for α-synuclein in tandem with the gene for the 434 repressor protein with an N-terminal His-tag and a caspase 7 cleavage site. Plasmids were transformed into BL21 (DE3) competent cells and grown at 37°C in 5x LB medium supplemented with 100 mg/L carbenicillin and grown until OD_600_~0.6. For each variant, including wild type, protein expression was induced with 0.5 mM isopropyl b-D-1-thiogalactopyranoside (IPTG) and then incubated for 6 hrs at 37°C. The cells were centrifuged for 30 min at 5000 rpm, and the pellet re-suspended in 8 M urea, 20 mM Tris, 20 mM imidazole, pH 8.0, sonicated on ice, followed by centrifugation at 20000 rpm for 30 mins. The supernatant was filtered and loaded on an affinity column (Ni Sepharose 6 Fast Flow, GE Healthcare), equilibrated with 20 mM Tris, 50 mM NaCl, 20 mM imidazole, 5% glycerol, pH 7.5, and eluted with the same buffer, but containing 250 mM imidazole. For removal of the His-tag as well as the co-expressed protein, the peptidase caspase 7 was added in a ratio of 1:100 (w/w), together with 20 mM 2-mercaptoethanol and was incubated further overnight at 4°C. Cleavage efficiency was verified with SDS-PAGE, and selected fractions were diluted 1:1 (v/v) with Milli-Q water. The sample was then loaded on a anion-exchange column (HiTrap Q FF, GE Healthcare) equilibrated with 20 mM Tris pH 8.0, and eluted with a linear NaCl gradient of 20 mM Tris, 1 M NaCl, pH 8.0. Finally, α-synuclein was run through a gel filtration column (HiLoad 16/60 Superdex 75, GE Healthcare), equilibrated with 50 mM ammonium carbonate. The α-synuclein concentration was determined using the absorption at 280 nm.

### Expression and purification of CsgE and CsgC

His-tagged CsgE was expressed and purified as described [24] with some modifications. Briefly, CsgE was expressed from pNH27 (gene encoding cytoplasmic CsgE inserted into the NcoI-BamHI sites of pET11d) in strain NEB 3016 in LB medium supplemented with 100 μg/ml ampicillin. Cells were grown to OD_600_ = 0.9 and induced for 3 h with IPTG. Cells were pelleted by centrifugation and re-suspended in lysis buffer (50 mM potassium phosphate, 200 mM NaCl, 0.1% Tween-20, 1 U/ml DNAse I). Cells were lysed by sonication and after centrifugation the supernatant was incubated with Ni-NTA resin (Sigma-Aldrich) at 4°C overnight. The nickel affinity beads were washed with lysis buffer, followed by a 10 mM imidazole, 50 mM potassium phosphate solution. His-tagged CsgE was eluted with a 250 mM imidazole, 50 mM potassium phosphate solution [23]. Finally, the protein was dialyzed against 50 mM potassium phosphate, 100 mM NaCl at 4°C overnight. His-tagged CsgC was purified as previously described [25] with some modifications. Briefly, CsgC was expressed from pET28a (gene encoding CsgC inserted into the NcoI-XhoI sites) in strain BL21 (DE3) in LB medium supplemented with 50 μg/ml kanamycin and 25 μg/ml chloramphenicol. Cells were grown to OD_600_ = 0.6, protein expression was induced with IPTG, and the cells were further grown over night. The cells were pelleted by centrifugation and re-suspended in lysis buffer (50 mM Tris, 300 mM NaCl, 10 mM imidazole, pH 7.8, 1 U/ml DNaseI). Cells were lysed using sonication and after centrifugation the supernatant was filtered and loaded on an affinity column (Ni Sepharose 6 Fast Flow, GE Healthcare) equilibrated with 50 mM Tris, 300 mM NaCl, 10 mM imidazole, pH 7.8, and eluted with 50 mM Tris, 300 mM NaCl, 250 mM imidazole, at pH 7.8. Next, the protein was run through a gel filtration column (HiLoad 16/60 Superdex 75, GE Healthcare), equilibrated with PBS (10 mM phosphate buffer, 140 mM NaCl, 2.7 mM KCl, pH 7.4).

### Thioflavin T (ThT) assay

α-synuclein amyloid formation assays (human, mouse, and variants) were conducted at 37°C with constant agitation with a 2 mm glass bead in each well (70 μM synuclein, 20 μM ThT in PBS) [10, 11] in the presence and absence of CsgC and CsgE concentrations as indicated in each case. Graphs are averages of at least 3 independent experiments. The emission intensities in the different reactions have been normalized (0–1) with respect to wild-type α-synuclein aggregation that was set to 1.

### Atomic force microscopy (AFM)

AFM measurements were performed on a BioScope Catalyst AFM (Bruker) in peak force mode in air at a resonance frequency of 70 kHz and a resolution of 256 × 256 pixels. Samples were diluted to approximately 5 μM with sterile filtered Milli-Q water and applied to freshly cleaved mica surface (Ted Pella), incubated for 15 min, washed 3 times with Milli-Q water and dried at room temperature.

### Fluorescence microscopy

Samples from completed ThT assays were diluted 1:10 with Milli-Q water and added to a glass slide and covered with cover slip before analyzed in a Zeiss Imager Z1 (Zen software). Representative images display only the FITC channel. The same exposure times was used for all images.

### Circular dichroism

Spectra were recorded on Jasco J-710 and J-810 spectropolarimeters at 20°C (0.1 cm quartz cuvette, 190–300 nm).

### NMR

NMR experiments were performed with a Bruker Avance III HD 850 MHz spectrometer equipped with a z-gradient cryoprobe using protein samples containing 5% D_2_O (v/v) in PBS at pH 7.4 and 10°C. The sample concentration was 70 μM for shaken/incubated α-synuclein samples, and 100 μM for non-shaken samples. All data processing was performed with NMRPipe [26] and data were analyzed in NMRView [27]. Integration and processing of diffusion data were made in TopSpin 3.2 and Microcal Origin 6.0. Translational diffusion data were obtained using pulsed field bipolar gradient stimulated echo experiments recorded at 10°C. Water suppression was achieved with pre-relaxation and WATERGATE. In total, 21 different gradient strengths were used for 6 ms, followed by a 100 ms recovery delay. The aliphatic region of α-synuclein (0–4.5 ppm) was integrated based on the 1D spectra, yielding signal amplitudes for each gradient. Calibration of the gradient strengths G was performed by a standard protocol [28]. The temperature was calibrated prior to the experiments by inserting a temperature probe into the sample compartment of the spectrometer. The initial signal amplitude I_(0)_ and the diffusion coefficient D was calculated according to the Stejskal-Tanner equation [29, 30], where Δ is the diffusion delay, ɣ is the gyromagnetic ratio, and δ the gradient pulse length:

$$\text{I}\left(\text{G}\right){=\text{I}}_{\left(0\right)}{}^{{\text{-G}}^{2}\gamma^{2}\delta^{2}\text{D}\left(\Delta \text{-}\frac{\delta }{3}\right)}$$ (1)

The diffusion coefficient was then converted into the hydrodynamic radius using the Stokes-Einstein equation [29], according to:

$${\text{R}}_{\text{h}}=\frac{k_{B}T}{6\pi \eta D}$$ (2)

Here, η is the dynamic viscosity of the solvent, R_h_ the hydrodynamic radius, k_B_ is Boltzmann’s constant, and T is the absolute temperature. A value of η = 1.307 cP was used for 5% D_2_O in PBS at 283.15 K [31].

## Results

**Fig 1:**
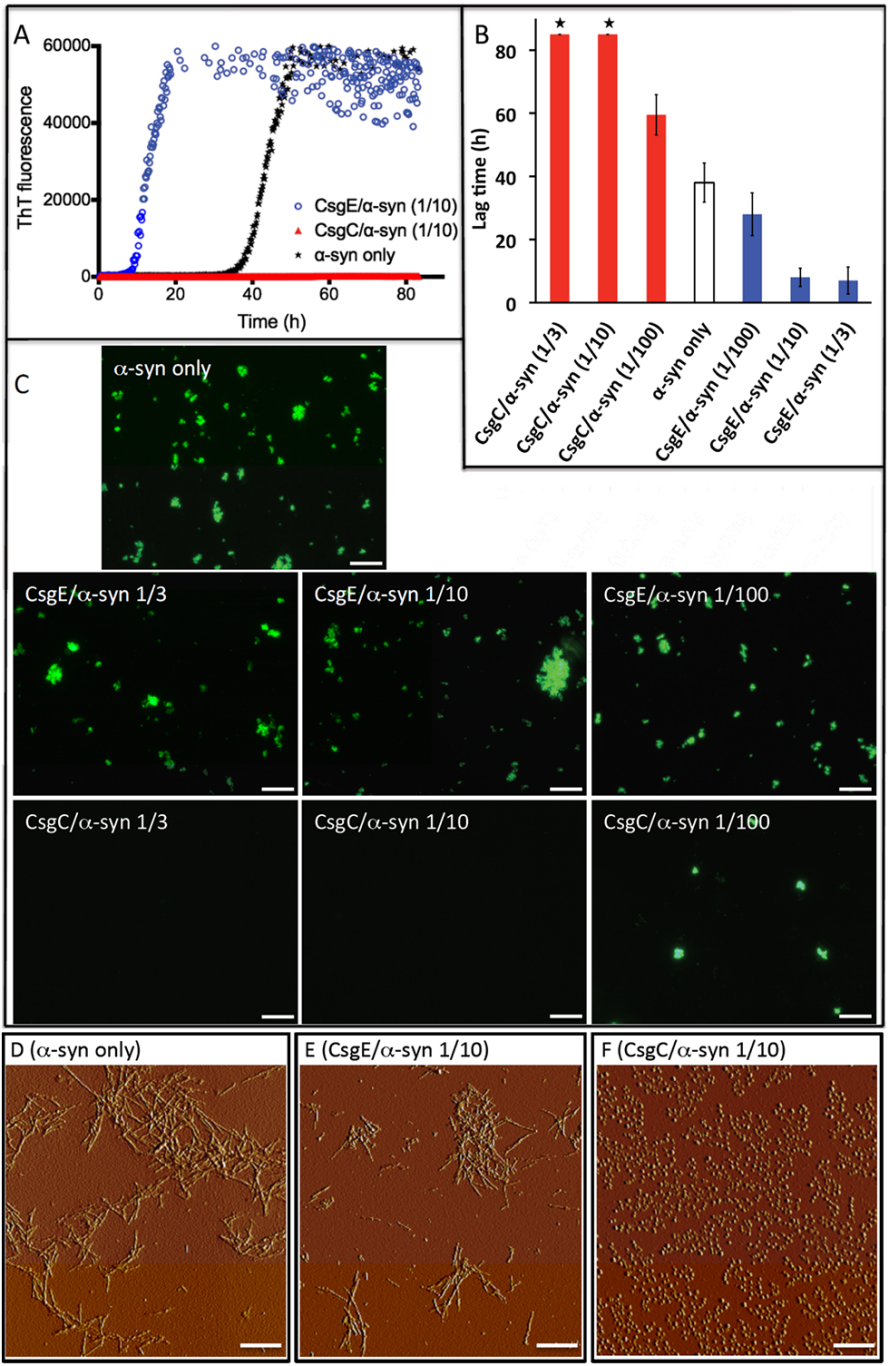
Aggregation of α-synuclein in the presence of CsgC and CsgE. **A.** ThT assay for α-synuclein aggregation with and without 1-to-10 molar ratio of CsgE:synuclein (red) or 1-to-10 molar ratio of CsgC:synuclein (blue). **B.** Bar graph showing the lag time for α-synuclein aggregation at 3 different ratios of CsgE and CsgC (1-to-3, 1-to-10, 1-to-100; 70 μM α-synuclein in all cases). ‘*’ Denotes no rise in ThT emission after 85 hrs. The error bars represent three experimental replicates. **C.** Fluorescence microscopy of end products of ThT assay for α-synuclein alone and for 1-to-3, 1-to-10, and 1-to-100 molar ratio of CsgE/CsgC-to-synuclein mixtures. Scale bar 100 μm. **D-F.** AFM images of end products after ThT experiments for α-synuclein alone (**D**), and in the presence of CsgE (**E**) and CsgC (**F**), as indicated. Scale bar 1 μm.

Amyloid fiber formation by α-synuclein can be monitored *in vitro* by Thioflavin T (ThT) fluorescence. When soluble monomeric α-synuclein is mixed with ThT and incubated at 37°C there is typically a lag phase of low fluorescence followed by a rapid increase in fluorescence indicative of amyloid fiber formation. In the lag phase, monomers assemble into smaller units that, upon reaching a critical concentration, induce rapid amyloid fiber formation and increased ThT emission [32]. When CsgE was added to α-synuclein aggregation reactions at a 1-to-10 molar ratio of CsgE to α-synuclein, the lag phase of α-synuclein amyloid formation was reduced by a factor of four (**Fig 1A**). When CsgE was added at a 1-to-3 molar ration with respect to α-synuclein, the lag phase of amyloid formation remained four-fold reduced. At a 1-to-100 molar ratio of CsgE to α-synuclein, however, the lag phase was only modestly affected (**Fig 1B**). Using fluorescence microscopy, we confirmed that ThT-bound aggregates were formed when α-synuclein was incubated alone and in the presence of CsgE at 1-to-3, 1-to-10 and 1-to-100 molar ratios (**Fig 1C**). Atomic force microscopy (AFM) analysis revealed amyloid-like fibrils when α-synuclein was incubated alone or in the presence of CsgE (**Fig 1D and 1E**). As evidenced from the presence of a precipitate upon centrifugation, amyloid fibers were present in the sample already at the time point (~18 h) when the ThT signal reached its maximum in the CsgE-α-synuclein sample. Taken together, CsgE appears to reduce the lag phase and accelerate the formation of α-synuclein amyloids that are macroscopically similar to those formed without CsgE.

The effect that CsgE had on α-synuclein amyloid formation was opposite to what was observed for CsgC [20]. When CsgC was added to α-synuclein in a 1-to-3 or 1-to-10 molar ratio of CsgC to α-synuclein, amyloid formation was completely inhibited for the entirety of the experiment (80 hrs) as measured by ThT fluorescence whereas at a 1-to100 molar ratio the lag time was increased by a factor of ~1.5 (**Fig 1A and 1B**). To verify that CsgC was preventing amyloid fiber formation and not simply interfering with ThT fluorescence, samples were analyzed by fluorescence and atomic force microscopy. Amyloid fibers were not observed when α-synuclein was incubated at 1-to-3 or 1-to-10 molar ratios with CsgC (**Fig 1C**) but when the molar ratio was lowered to 1-to-100 (CsgC to α-synuclein), fluorescent clusters became visible (**Fig 1C**). AFM revealed the presence of spherical oligomers when α-synuclein was incubated at a 1-to-10 molar ratio of CsgC to α-synuclein (**Fig 1F**). Since pre-fibrillar oligomers do not promote increased ThT emission [32], the AFM observation is in agreement with the ThT results for the CsgC-α-synculein mixtures.

**Fig 2:**
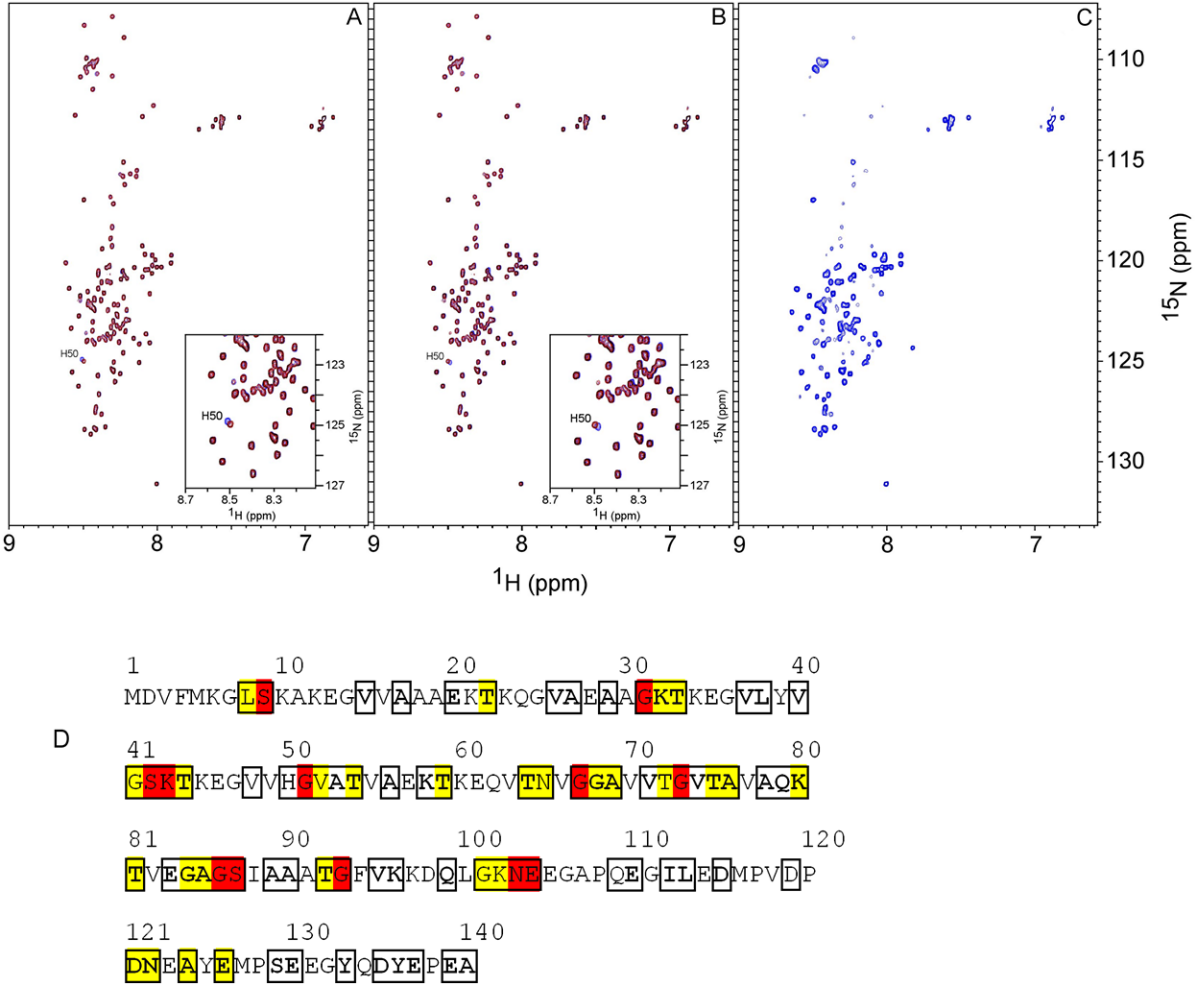
Solution NMR of CsgC/CsgE interactions with ^15^N labeled α-synuclein. ^1^H-^15^N HSQC spectra at 10°C for 100 μM α-synuclein alone (red data in all panels) and upon addition of a 1-to-1 molar ratio of CsgC (**A**, blue) and CsgE (**B**, blue), and for a 1-to-5 molar ratio sample of CsgC and α-synuclein that had been shaken at 37°C for 48 h (**C,** blue). The data shown in **A** and **B** demonstrate that blue and red signals overlap except for His50 (**Insets** in **A** and **B**) and these spectra did not change over the course of three days. The visible chemical shifts in **C** were analyzed by NMR diffusion experiments to obtain an estimate of the molecular size. **D.** Analysis of perturbed residues in α-synuclein in the incubated CsgC-synuclein sample, based on the ^1^H-^15^N HSQC peak intensities in **C** and reported assignments [33, 34]. Boxed residues represent chemical shifts assigned to specific residues (78 of the 140 residues were identified and used for the analysis). After shaking, 38 residues disappeared or broadened severely in the new species as judged from the peak intensities. Residues that broadened beyond detection are shown in red, and residues that lost > 90% of the original intensity are shown in yellow. Marked in bold are residues that show no apparent chemical shift change (Δω < 0.02 ppm, calculated as Δω = |0.2Δ^15^N+Δ^1^H|).

To identify the nature of the interactions between CsgC/CsgE and α-synuclein we turned to nuclear magnetic resonance (NMR). When CsgC or CsgE (unlabeled) were mixed (without shaking) with ^15^N-labeled α-synuclein, α-synuclein remained monomeric and unfolded for at least three days (**Fig 2A and 2B**). In the presence of CsgC or CsgE, a single chemical shift change was observed in the α-synuclein spectrum, corresponding to His50 (**Insets, Fig 2A and 2B**). To assess potential pH effects on the histidine upon chaperone addition, we determined that the pH values between the samples differed by up to 0.06 pH units. Although small, this difference may have caused the small observed perturbations of His50. The overall lack of chemical shift changes upon the addition of bacterial proteins indicated that CsgC and CsgE did not stably interact with α-synuclein monomers. In accord with the NMR data, we could not detect an enthalpic binding event between either CsgE or CsgC and α-synuclein monomers using isothermal titration calorimetry (ITC) at 20°C (data not shown). ^1^H-^15^N HSQC experiments and calorimetric titrations could not be performed at 37°C due to poor spectral quality (2D NMR) and increased background heats (ITC).

We also used NMR to characterize the biophysical state of ^15^N-labeled α-synuclein in the presence of CsgC after agitation at 37°C for 48 hours. When a sample with CsgC and α-synuclein had been shaken on the plate reader (ThT data for NMR sample; **S1 Fig**), the ^1^H-^15^N HSQC spectrum of α-synuclein was affected relative to α-synuclein alone. The spectrum after incubation with CsgC exhibited severe line broadening of many α-synuclein peaks and many had fully disappeared (**Fig 2C**). The affected residues were distributed throughout the α-synuclein polypeptide but most were concentrated to the core segment (**Fig 2C**). Out of 78 identified peaks in α-synuclein, 38 disappeared or were reduced to 10% of the original intensity in the incubated sample. 32 of the 38 disappearing peaks corresponded to residues within the 30–104 region of α-synuclein. The observed chemical shift changes are displayed on the α-synuclein primary sequence in **Fig 2D**. Taken together, the NMR data demonstrated that a new species was formed that was large enough in size to cause peaks to disappear in the ^1^H-^15^N HSQC spectrum (slow tumbling) and only its unordered parts (that tumble faster in solution and can be detected by NMR) were visible in the spectrum.

The diffusion constant and hydrodynamic radius for the α-synuclein species formed upon shaking with CsgC was determined from integration of α-synuclein signals in the methyl region in the 1D spectra. The estimated hydrodynamic radius, ~4 nm, corresponded to an ensemble of α-synuclein species larger than the monomer (**S2 Fig**) as the monomer hydrodynamic radius at the same condition was previously reported to be ~2.8 nm [10]. Thus, consistent with the AFM data (**Fig 1E**), CsgC is directing α-synuclein into an ogliomeric state that does not appear to be on pathway to amyloid fiber formation.

**Fig 3:**
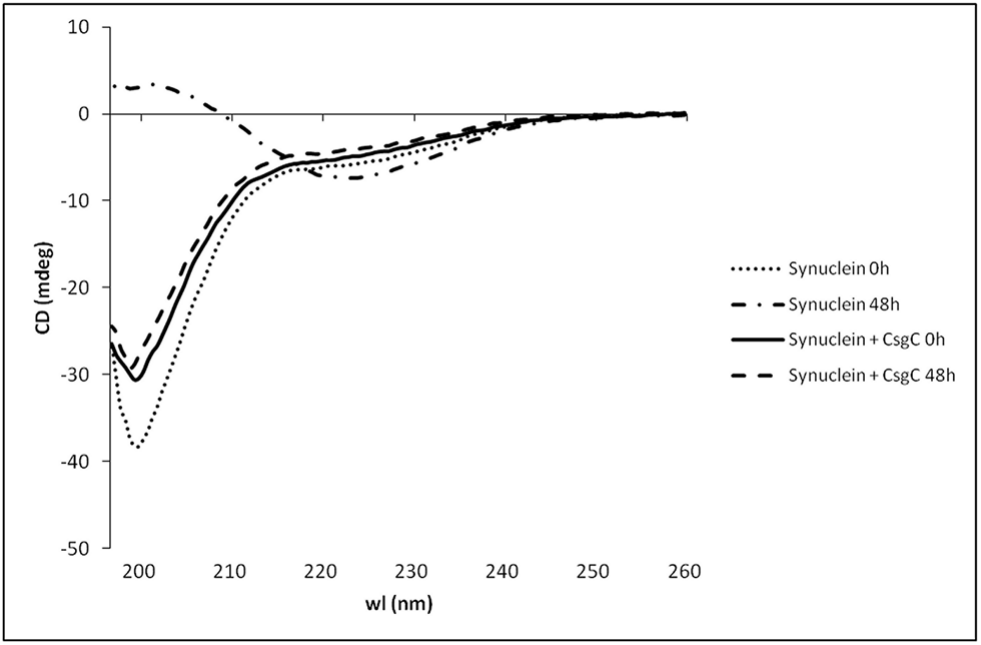
Secondary structure of CsgC-induced oligomers. Far-UV CD spectra for 70 μM monomeric α-synuclein alone, and after shaking at 37°C for 48 h with and without 14 μM CsgC. The contribution from CsgC to the CD signal has been subtracted (CsgC shaken alone for 48 h did not result in any change of CD signal, data not shown). Whereas monomeric α-synuclein has random-coil like secondary structure, α-synuclein adopts β-sheet structure after incubation as a result of amyloid fiber formation. In the presence of CsgC, α-synuclein remains random-coil also after 48 h of shaking at 37°C.

By comparing 1D spectra of CsgC alone and the CsgC and α-synuclein mixture shaken at 37°C, it was evident from the similarity in line-shape of isolated CsgC peaks that CsgC remained free in solution in the mixture (data not shown). Thus, the NMR data indicated that transient CsgC interactions promoted the formation of inert α-synuclein oligomers under fibril forming conditions and CsgC itself was not incorporated into these oligomers. Based on far-UV CD data, the CsgC-induced α-synuclein oligomers (that appeared spherical according to AFM) had random coil secondary structure (**Fig 3**).

## Discussion

Because assembly and amyloid formation of α-synuclein play key roles in Parkinson’s disease, there has been a concerted effort to find small-molecule effector molecules that redirect α-synuclein to less toxic molecular species [7, 8, 10, 11]. The general heat shock chaperones Hsp70 and Hsp90 can prevent α-synuclein amyloid formation *in vitro* [35], suggesting that triggering of cellular responses to protein misfolding may be a way to temper disease progression. There are also multiple ‘functional’ amyloid assembly systems, and some of these feature chaperone-like proteins that help control amyloid formation so that it takes place at the correct time and place [12, 13]. The bacterial amyloid called curli has at least two chaperone-like proteins that discourage the aggregation of the major amyloid forming protein, CsgA, thus preventing aggregation and fiber formation in the periplasm [20, 24]. Since amyloids have a common cross-β structure, we hypothesized that these bacterial chaperones might also modulate human α-synuclein aggregation, as we had previously discovered that certain small molecule peptidomimetics cross-reacted with CsgA and α-synuclein [10, 11, 21]. The possibility of a small molecule or protein acting as both an accelerator and inhibitor depending on the amyloidogenic target protein emphasizes the importance of cross-reactivity tests when developing new amyloid effectors.

The bacterial chaperone-like proteins CsgC and CsgE inhibit amyloid formation by the curli protein CsgA and CsgC inhibits α-synuclein amyloid formation [20, 21, 24]. Here, we show that in contrast to CsgC, CsgE accelerates α-synuclein amyloid formation *in vitro* (**Fig 1**). Although the fact that CsgE inhibits CsgA amyloids but accelerates α-synuclein amyloid formation seems contradictory, we observed the exact same trend with a 2-pyridone called FN075, which inhibits CsgA [36], but accelerates α-synuclein [10] amyloid formation.

We found that both CsgE and CsgC mediate their modulation of α-synuclein amyloid formation using transient interactions since there is no change in the ^1^H-^15^N HSQC NMR spectrum for α-synuclein upon their addition (**Fig 2A and 2B**). That the CsgE and CsgC interactions are found to be transient provide an explanation for the ability of both bacterial chaperones to affect α-synuclein amyloid formation at sub-stoichiometric concentrations (**Fig 1B**). At aggregation-promoting conditions, the CgsC interactions with α-synuclein resulted in random-coil structured α-synuclein oligomers (**Fig 2C, Fig 3**) that did not proceed to amyloid fibers (**Fig 1F**). Based on chemical shift analysis, these oligomers appeared to have floppy C- and N-termini (**Fig 2D**).

Our data suggest that transient interactions between bacterial chaperones and α-synuclein can result in opposite effects on α-synuclein amyloid formation. This may be rationalized by considering that intra-protein interactions between the amyloidogenic NAC and C-terminal domains of α-synuclein are thought to normally temper amyloid formation [37]. If CsgE transiently interacts such that it facilitates the release of default interactions between the C-terminal and NAC domains, and thereby the NAC exposure increases, this may promote amyloid formation. Alternatively, because CsgE appears to be an oligomer [23], it may interact with several α-synuclein polypeptides simultaneously, acting as a collecting point promoting aggregation-prone interactions. When α-synuclein was mutated in the 98–105 amino acid region, the resulting α-synuclein variants formed amyloids much faster than wild-type α-synuclein, and CsgE was not able to accelerate the reaction anymore (**S3 Fig**). This implies that either the mutated region is a direct interaction site or the introduced mutations reduce the same kinetic barrier as the presence of CgsE does. For the inhibitor CsgC, instead, transient interactions may facilitate additional intra-protein contacts shielding the NAC domain, thereby blocking amyloid formation. Mechanistic studies with sensitive probes able to catch transient interactions and conformational changes in the α-synuclein polypeptide will be required to resolve these issues in the future.

In conclusion, we discovered that CsgE and CsgC transiently interact with α-synuclein, which results in acceleration (CsgE) or inhibition (CsgC) of amyloid formation. In the case of CsgC inhibition of CsgA amyloid formation, it was suggested that CsgC acts transiently on small pre-amyloid assemblies of CsgA blocking them from crossing the barrier towards amyloid fibers [20]. This mechanistic concept is similar to what we propose here for CsgE and CsgC interactions with α-synuclein. In analogy to our CsgC results, non-canonical (*i*.*e*., transient) interactions with α-synuclein monomers have been reported for DnaK that resulted in amyloid inhibition [38] and the anti-aggregation activity by antibodies on α-synuclein was reported to take place via a pseudo-catalytic mechanism [39]. In similarity to the CsgE results, FKBP12 was suggested to enhance α-synuclein aggregation via a catalytic mechanism involving transient interactions [40]. Moreover, two different variants of β2-microglobulin were reported to interact via similar surfaces but resulting in either inhibition or promotion of fibril formation [41]. We propose that weak transient interactions that in a catalytic fashion shift the conformational ensembles of amyloidogenic polypeptides represent an efficient amyloid modulator mechanism.

Humans harbor a vast ensemble of microbes in the gut [42] and both due to excretion and lysis of bacterial cells there will be bacterial proteins in the circulation system. Parkinson’s disease has been proposed to spread via the peripheral nervous system from the gut [43, 44] and one may speculate that direct interactions with bacterial proteins may influence disease progression *in vivo*. Certainly, more work is needed to appreciate how the microbiome affects the trajectories of neurological diseases.

## Supporting Information

S1 Fig ThT aggregation data for the sample used in the NMR experiment in Fig 2C. The original CsgC-synuclein sample was divided into two aliquots and put on the plate reader. For one of the samples ThT was added to detect aggregation; the other was incubated without ThT to be used for NMR. Since ^15^N-labeled α-synuclein aggregated rather slow in this experiment, the NMR sample was used after 70 h of incubation. As a control, CsgE was included in this experiment to demonstrate that ^15^N-labeling of α-synuclein did not perturb the ability of CsgE to shorten the lag time of amyloid aggregation.
(TIF)

S2 Fig Integrated 1D data for analysis of CsgC-induced α-synuclein oligomers. The figure shows the bi-exponential fit of DOSY data for CsgC-synuclein (1-to-5 molar ratio) that was shaken at 37°C for 48 h (NMR data shown in **Fig 2C**). The data corresponds to a sphere with a R_H_ of 4.0 nm.
(TIF)

S3 Fig Amyloid formation of fast-aggregating α-synuclein variants in the presence of CsgE. ThT assay for α-synuclein variants 6Ala (**A**) and random (**B**) with 1-to-5 molar ratio of CsgE to α-synuclein (red) and without CsgE (black). In the 6Ala variant, D98, Q99, G101, K102, N103 and E105 are exchanged for Ala in the _98_DQLGKNEE_105_ stretch of the α-synuclein sequence. In the random variant, the whole motif is exchanged for SQGAYGNTA (lacking charges, originally selected from a motif in CsgB).
(TIF)
