# Supplementary figures and images for: Bacterial Chaperones CsgE and CsgC Differentially Modulate Human α-Synuclein Amyloid Formation via Transient Contacts
Source: PLoS One. 2015 Oct 14;10(10):e0140194. doi: 10.1371/journal.pone.0140194 (PMC4605646; doi:10.1371/journal.pone.0140194)

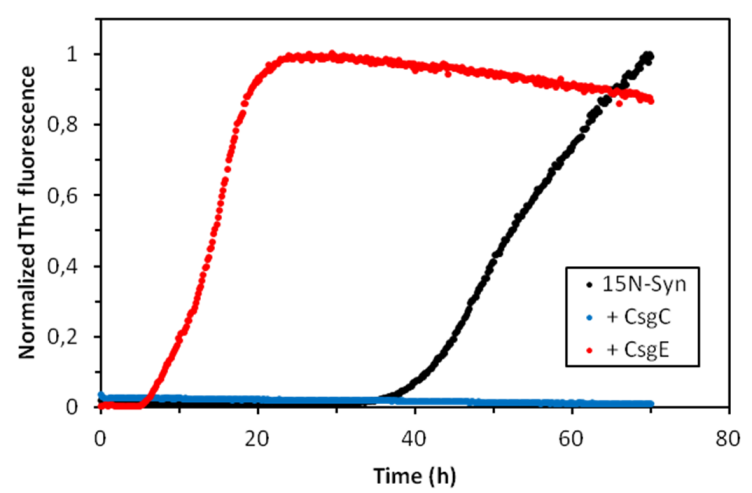

Supplement: S1 Fig — The original CsgC-synuclein sample was divided into two aliquots and put on the plate reader. For one of the samples ThT was added to detect aggregation; the other was incubated without ThT to be used for NMR. Since 15N-labeled α-synuclein aggregated rather slow in this experiment, the NMR sample was used after 70 h of incubation. As a control, CsgE was included in this experiment to demonstrate that 15N-labeling of α-synuclein did not perturb the ability of CsgE to shorten the lag time of amyloid aggregation. (TIF) [file pone.0140194.s001.tif]

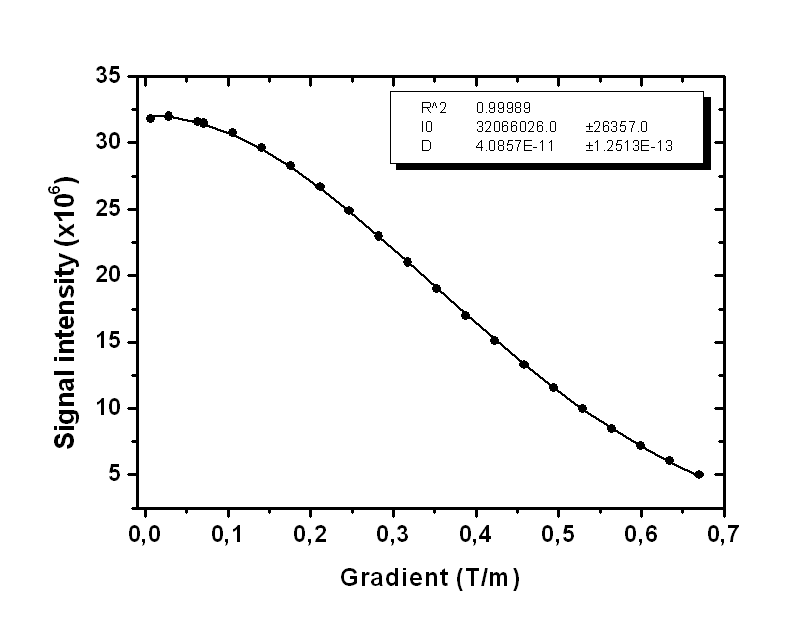

Supplement: S2 Fig — The figure shows the bi-exponential fit of DOSY data for CsgC-synuclein (1-to-5 molar ratio) that was shaken at 37°C for 48 h (NMR data shown in Fig 2C). The data corresponds to a sphere with a RH of 4.0 nm. (TIF) [file pone.0140194.s002.tif]

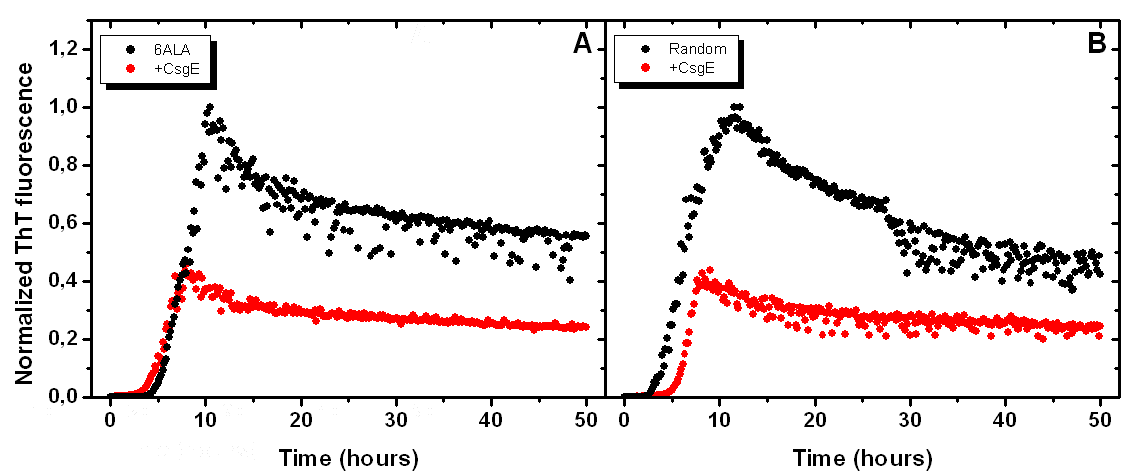

Supplement: S3 Fig — ThT assay for α-synuclein variants 6Ala (A) and random (B) with 1-to-5 molar ratio of CsgE to α-synuclein (red) and without CsgE (black). In the 6Ala variant, D98, Q99, G101, K102, N103 and E105 are exchanged for Ala in the 98DQLGKNEE105 stretch of the α-synuclein sequence. In the random variant, the whole motif is exchanged for SQGAYGNTA (lacking charges, originally selected from a motif in CsgB). (TIF) [file pone.0140194.s003.tif]
